# Clinical Experience with Adrenalin

**Published:** 1901-07

**Authors:** 


### CLINICAL EXPERIENCE WITH ADRENALIN.

Emil Mayer, of New York, (*Phil. Med. Jour.*,) says that Dr. Jokichi Takamine undertook the task of isolating the active principle of the suprarenal gland and obtained a substance in stable and pure crystalline form, which raises the blood pressure, and which he named adrenalin.

The author has used solutions of adrenalin chloride, 1 to 1,000, 1 to 5,000 and 1 to 10,000; his cases were all rhinological. Blanching of tissues followed the application of the strongest of these solutions in a few seconds, and was very thorough. In no instance was there any constitutional disturbance. He has employed no suprarenal extract since, for any purpose whatever.

Thirty-five cases are reported, showing that the usual effect of the aqueous extract of the suprarenal gland was obtained. A few operative cases bled freely, but in every instance, the hemorrhage was promptly checked by a second application of adrenalin. The adrenalin was used not only as a hemostatic, but for the relief of nasal congestion, as a diagnostic aid, and for the continuous treatment of acute inflammatory affections of the accessory sinuses.

The author arrives at the following conclusions: (1) adrenalin solutions supply every indication for which the aqueous extract has been used; (2) they are sterile; (3) they keep indefinitely (4) solutions, 1 to 1,000 are strong enough for operative work, and 1 to 5,000 and 1 to 10,000 for local medication; (5) they may be used with safety.

